# Ethylene Is Not Responsible for Phytochrome-Mediated Apical Hook Exaggeration in Tomato

**DOI:** 10.3389/fpls.2016.01756

**Published:** 2016-11-23

**Authors:** Miki Takahashi-Asami, Chizuko Shichijo, Seiji Tsurumi, Tohru Hashimoto

**Affiliations:** ^1^Plant Physiology, Department of Biology, Graduate School of Science, Kobe UniversityKobe, Japan; ^2^Center for Supports to Research and Education Activities, Kobe UniversityKobe, Japan; ^3^Uozaki Life Science LaboratoryKobe, Japan

**Keywords:** apical hook, auxin, ethylene, hypocotyl, light-induced hook exaggeration (LIHE), phytochrome, tomato (*Solanum lycopersicum*)

## Abstract

The apical hook of tomato seedlings is exaggerated by phytochrome actions, while in other species such as bean, pea and *Arabidopsis*, the hook is exaggerated by ethylene and opens by phytochrome actions. The present study was aimed to clarify mainly whether ethylene is responsible for the phytochrome-mediated hook exaggeration of tomato seedlings. Dark-grown 5-day-old seedlings were subjected to various ways of ethylene application in the dark as well as under the actions of red (R) or far-red light (FR). The ethylene emitted by seedlings was also quantified relative to hook exaggeration. The results show: Ambient ethylene, up-to about 1.0 μL L^-1^, suppressed (opened) the hooks formed in the dark as well as the ones exaggerated by R or FR, while at 3.0–10 μL L^-1^ it enhanced (closed) the hook only slightly as compared with the most-suppressed level at about 1.0 μL L^-1^. Treatment with 1-aminocyclopropane-1-carboxylic acid (ACC), the immediate precursor of ethylene biosynthesis, did not enhance the hook, only mimicking the suppressive effects of ambient ethylene. The biosynthesis inhibitor, CoCl_2_ or aminoethoxyvinylglycine, enhanced hook curvature, and the enhancement was canceled by supplement of ethylene below 1.0 μL L^-1^. Auxin transport inhibitor, N-1-naphthylphthalamic acid, by contrast, suppressed curvature markedly without altering ethylene emission. The effects of the above-stated treatments did not differentiate qualitatively among the R-, FR-irradiated seedlings and dark control so as to explain phytochrome-mediated hook exaggeration. In addition, ethylene emission by seedlings was affected neither by R nor FR at such fluences as to cause hook exaggeration. In conclusion, (1) ethylene suppresses not only the light-exaggerated hook, but also the dark-formed one; (2) ethylene emission is not affected by R or FR, and also not correlated with the hook exaggerations; thus ethylene is not responsible for the hook exaggeration in tomato; and (3) auxin is essential for the maintenance and development of the hook in tomato as is the case in other species lacking phytochrome-mediated hook exaggeration. A possible mechanism of phytochrome action for hook exaggeration is discussed.

## Introduction

Through a long history of research it has been widely accepted that the apical hook is formed in the dark and opens in the light. Recently, however, this notion was shown not to apply to all species. In tomato and some other dicotyledonous species characterized by seeds bearing the hard-to-split tough seed coat and the rich endosperm, the apical hook is markedly exaggerated by exposure to light, mediated by phytochromes through the very low, low and high irradiance responses, and hence activated by red (R) as well as far-red light (FR). The hook exaggeration and its subsequent opening motion facilitate the release of the seed coat, and contribute to the survival of the seedling above the ground (Shichijo et al., 2010a,b; Shichijo and Hashimoto, 2013).

So far in the past, on the other hand, many works clarified with a range of dicotyledonous species represented by pea, bean, and recently *Arabidopsis* that the apical hook is formed in the dark and opens in the light, mediated by phytochrome, and that it is enhanced by ambient ethylene, and further, exaggerated at higher concentrations (Withrow et al., 1957; Goeschl et al., 1967; Kang and Ray, 1969; Powell and Morgan, 1980; Britz and Galston, 1982; Liscum and Hangarter, 1993a,b; Li et al., 2004; Abbas et al., 2013; Mazzella et al., 2014; Willige and Chory, 2015; Žádníková et al., 2015). It was also shown that ethylene emission from seedlings is enhanced by mechanical stresses, and ethylene-induced hook exaggeration is suppressed by light through phytochrome mediation (Harpham et al., 1991; Ecker, 1995; Lehman et al., 1996; Raz and Ecker, 1999; Knee et al., 2000; Zhong et al., 2014; Shi et al., 2016).

Compared with the hook exaggeration in *Arabidopsis* referred to above, the case in tomato is very similar in shape, but different in that the hook is enhanced by light. Hence it is tempting to examine with tomato seedlings whether ethylene causes hook exaggeration, and whether ethylene emission is increased by light. In the present study these questions are examined by supplement of ethylene gas and biosynthetic precursor of ethylene, suppression of endogenous ethylene level by biosynthetic inhibitors and hindrance of auxin polar transport by its inhibitor as well as quantification and comparison of endogenous ethylene evolved under R and FR as well as in the dark. A novel action of ethylene in the apical hook movement discovered during these tests will be described, and a possible mechanism of phytochrome-mediated apical hook exaggeration will be proposed.

## Materials and Methods

### Culture Bottle and Its Cap-Loose- and Cap-Tight Modes

**FIGURE 1:**
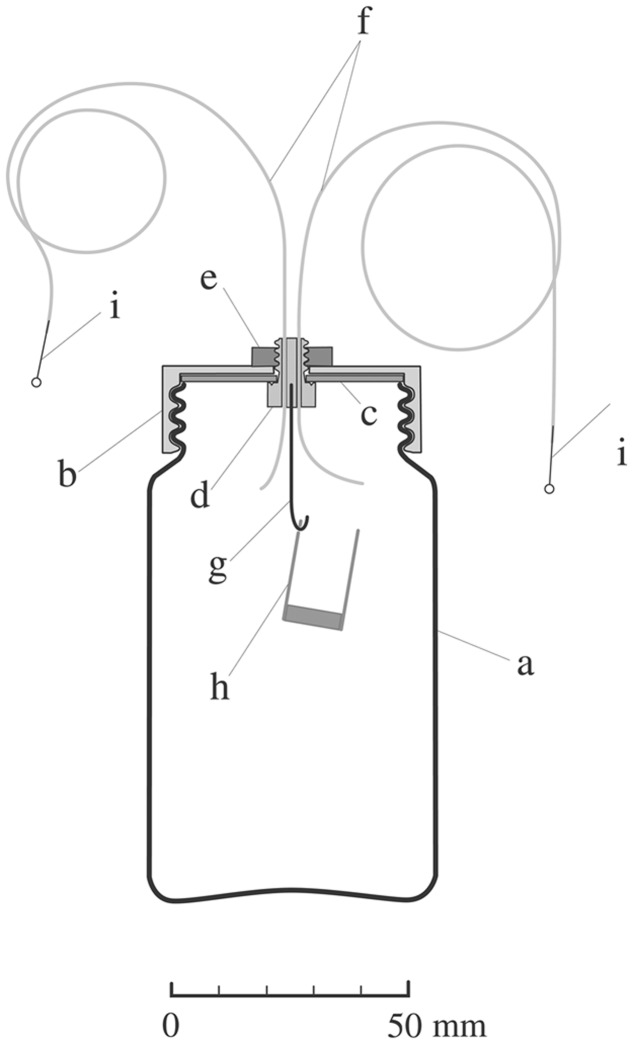
**Diagram of a culture bottle in the cap-tight mode devised for ethylene treatment and quantification.** A glass bottle (a), 62 mm × 110 mm (height), 48 mm in mouth diameter, 236 ml in volume equipped with a cap for the cap-tight mode which is composed of a TPX screw cap (b), 1.5-mm thick Teflon-coated packing sheet (c) (Top No. 588-08; Sogo Laboratory Glass Works Co., Kyoto, Japan), a Teflon bolt (d) with two holes for inlet and outlet tubes, an M6 stainless steel nut (e), inlet and outlet tubes (f), 300 and 500 mm long, 1.0 mm od., 0.5 mm id. (PTFE tube; Chukoh Chemical Industries, Ltd., Tokyo, Japan), Aluminum hook (g), CO_2_-absorbent container (h), 15 mm in diameter, 25 mm in height, and stopper pins (i), 0.5 mm in diameter.

In the present investigation, transparent glass bottles, 62 mm in diameter, 110 mm in height and 48 mm in bottle mouth diameter (**Figure 1**), served throughout for culturing seedlings and performing experiments in cap-loose and cap-tight modes. The cap-loose mode denotes the conditions that an ordinary plastic cap was screwed loosely on the bottle mouth so as to allow air flow freely, and the cap-tight mode means that the ordinary cap was replaced by another type of a cap equipped with inlet and outlet tubes and other necessities, and screwed tightly so as not to allow air to pass (**Figure 1**).

In assembling the cap for the cap-tight mode, special care was spent to prevent air leak through the cap assembly. The contact surfaces between the packing sheet (c) and the Teflon bolt (d) and between the Teflon bolt (d) and the inlet- and outlet-tubes (f) were pasted with fluorochemical lubricating grease (DEMUNUM GREASE, Daikin Industries, Ltd., Osaka, Japan). When screwing the cap (b) to the bottle (a), the bottle mouth edge was moistened with a drop of water to secure airtightness. Bottles thus prepared were examined for ethylene preservation for 4 days, and only cap-bottle combinations which preserved ethylene more than 95% were used.

### Plant Materials and Growth Conditions

Seeds of tomato (*Solanum lycopersicum* L.), cvs. Ponte-Rosa, Sekaiichi and Seikoh No.17, were purchased, respectively, from Ishihara Seed Co. (Sakai, Japan), Marutane Seed Co. (Kyoto, Japan) and Watanabe Seed Co. (Misatomachi, Miyagi, Japan). The three cultivars behaved similarly in phytochrome-mediated hook exaggeration (Shichijo et al., 2010a,b; Shichijo and Hashimoto, 2013), and were used arbitrarily in the present experiments. Seeds were sterilized for 10–20 min with 25-times diluted solution of commercial bleach, followed by thorough washing with running tap water for 30–90 min, and soaked in sterilized distilled water until full imbibition. Thirty to 40 seeds thus treated were germinated on a layer of 60-mm filter paper moistened with 3.5 or 4 ml of distilled water in culture bottles (**Figure 1**) and seedlings were grown at 25.5 ± 0.5°C throughout. In experiments where ethylene was quantified, the same number of seeds were sown in each culture bottle. Reagent solutions were applied to the filter paper instead of distilled water before sowing or sprayed to seedlings after grown for 5 days in culture bottles.

### Light Sources and Irradiation

The red light (R) sources used were R_LED_ (λ_max_, 658 nm; half-bandwidth, 26 nm) and R_FL_ (λ_max_, 660 nm; half-bandwidth, 20 nm); far-red light (FR) sources were FR_LED_ (λ_max_, 746 nm; half-bandwidth, 28 nm) and FR_FL_ (λ_max_, 766 nm; half-bandwidth, 72 nm). The details of these light sources were already described (Shichijo et al., 1993, 2001). Photon fluence rate was determined with an Optical-Power-Meter (TQ8210; Advantest, Tokyo, Japan). Irradiations were made from above, unilaterally or bilaterally. Since a single pulse of R or FR is enough to cause hook exaggerations, mediated by very low and low fluence responses of phytochrome, and allows free selection of an appropriate time-point for irradiation according to its purpose, a single pulse irradiation has been adopted as the standard, and where needed, the irradiation period was extended accordingly.

### Chemical Reagents

Ethylene gas (99.5%) was purchased from GL Sciences, Inc. Japan. 1-Aminocyclopropane-1-carboxylic acid (ACC) and aminoethoxyvinylglycine (AVG) were from Sigma–Aldrich through Hirose kagaku, Ltd, Kobe, Japan; cobalt chloride (CoCl_2_) and N-(1-naphthyl)phthalamic acid (NPA), from Wako Pure Chemical Industries, Osaka, Japan and Tokyo Chemical Industry, Tokyo, Japan, respectively. Ethylene was diluted with air, and the other reagents were dissolved in distilled water.

### Determination of Hook Curvature and Hypocotyl Height

Hook curvature was quantified by the angle formed by extended lines of the main hypocotyl axis and the apical axis, defining as zero degree when the apical hook is straightened up. The hypocotyl height designates the length from the highest point of hook to the base of the hypocotyl. These are in accordance with Shichijo et al. (2010a).

### Supplement of Ethylene

For applying and quantifying ethylene, culture bottles in the cap-tight mode were used (**Figure 1**). When growing seedlings in culture bottles of the cap-tight mode, the evolving CO_2_ was absorbed by the CO_2_-absorbent container (h in **Figure 1**) where 1.5 ml of 1 M NaOH is added to 0.1 g absorbent cotton.

To fill a definite concentration of ethylene in a culture bottle of the cap-tight mode, 1 ml of prescribed higher concentrations of ethylene/air mixture was injected so as to result in the required concentration when it dispersed within the bottle. A 1-ml empty syringe with a needle (od = 0.5 mm) was inserted into the outlet tubes (f in **Figure 1**) as the air receiver, and another same sized syringe filled with the prescribed concentration of ethylene/air mixture was similarly inserted in the inlet tube (f in **Figure 1**) as the donor syringe. As the receiver syringe sucked up air from the bottle, the donor syringe pressed the ethylene/air mixture into the bottle. After the ethylene injection was completed, the outlet and inlet tubes were closed with stopping pins (i in **Figure 1**).

### Quantification of Ethylene

To determine the ethylene concentrations in culture bottles, gas-sampling was made similarly by concerted motions of two syringes, one of which was filled with ethylene-free air of the same volume as that of the sample air to be taken with the other empty syringe. In this way, sample air of 1 or 2 ml was taken out and injected into a gas chromatograph (GC-14A; Shimadzu Co., Kyoto) equipped with a 100 cm × 0.26 cm active alumina (60–80 mesh) column (Gasukuro Kogyo Inc., Tokyo, Japan) and a flame ionization detector. In some experiments (**Figures 7** and **8**) ethylene emission rate was calculated with the equation (ethylene concentration × bottle capacity)/(Fresh weight of seedlings × incubation period).

### Statistical Analysis

Statistical significance was determined by the *post hoc* analysis, especially Tukey’s test, after one-way ANOVA by means of the software KaleidaGraph (Synergy Software, Inc., Reading, PA, USA), and has been indicated by asterisks, pound marks or alphabets in the vicinity of relevant data points or on the top of histogram bars to be compared in Figures.

## Results

### Effect of Ambient Ethylene on the Hook Curvatures Formed in the Dark as well as Exaggerated under Red and Far-Red Light

**FIGURE 2:**
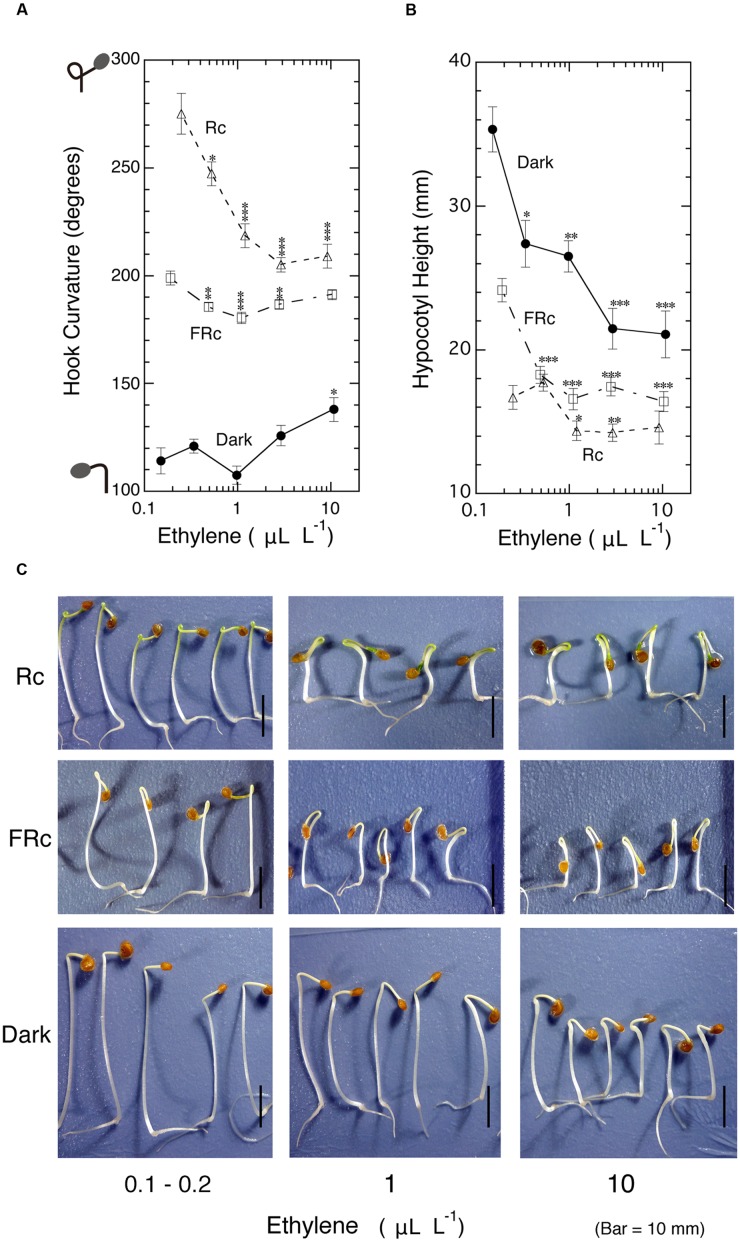
**Effects of ambient ethylene on apical hook curvature of dark-grown seedlings of tomato under Rc, FRc or in the dark.** After grown from seeds in the cap-loose mode in the dark for 5 days, seedlings were shifted to the cap-tight mode and exposed to various concentrations of ethylene and continuous R (Rc), FR (FRc) or kept in the dark for 50–56 h until the end of experiment, where final concentrations of ethylene for plotting data in **(A)** and **(B)**, hook curvature **(A)** and hypocotyl height **(B)** were determined, and the representative seedlings were photographed **(C)**. Supplemented ethylene concentrations: null, 0.3, 1.0, 3.0, and 10 μL L^-1^; Rc: R_FL_, 17 μmol m^-2^ s^-1^; FRc: FR_FL_, 14 μmol m^-2^ s^-1^. Data points: mean ± SE (*n* = 19–51) for hook angle and hypocotyl height. Statistical significance, respectively, at ^∗^*P* < 0.01, ^∗∗^*P* < 0.005, ^∗∗∗^*P* < 0.0001 as compared with the values at the lowest ethylene concentration (null supplemented ethylene) within each light condition. cv. Ponte-Rosa.

To examine whether ethylene causes the exaggeration of the apical hook instead of light, dark-grown 5-day-old tomato seedlings were exposed to various concentrations of ambient ethylene in culture bottles in the dark. As the standard of hook exaggerations mediated by very low and low fluence responses as well as high irradiance response of phytochrome (Shichijo et al., 2010a), seedlings were, without addition of exogenous ethylene, irradiated with continuous red (Rc) and far-red light (FRc) in place of pulsed light. In parallel, effects of ethylene on hook under such irradiated conditions were also examined (**Figure 2A**). In the present study, such low concentrations of ethylene as endogenous ethylene also matter, the concentrations in the culture bottles were determined at the end of experiments and used as ambient ethylene concentration to plot the hook response in this Figure and others. In the dark, ethylene suppressed (i.e., opened) hook curvature in the neighborhood of 1.0 μL L^-1^, and as its concentration increased beyond 1.0 μL L^-1^, the gas enhanced (i.e., closed) the hook, but even at 10 μL L^-1^, it could not intensify the curvature to the level of the hooks exaggerated by either Rc or FRc in the absence of supplementary ethylene (left end of the curves). As seen in the photos at 10 μL L^-1^ (**Figure 2C**), the toxicity of high concentration ethylene, i.e., the severe growth inhibition of the hypocotyl and the swelling at the part just below the hook, implies that no further enhancement of the hook was possible even by further raising ethylene concentration.

The hook exaggerated by Rc was suppressed by ethylene more markedly than the hook suppression in the dark. Similar suppression was also observed under FRc to lesser extents (**Figure 2A**). In both cases under light the enhancement of the hook angle at the higher ethylene concentration ranging from 3.0 to 10 μL L^-1^ was small in ratios compared with that observed in the dark. The suppression of hypocotyl height (**Figure 2B**) as well as the appearance of the seedlings (**Figure 2C**) shows that the ambient ethylene worked normally.

### Effect of Endogenous Ethylene Supplied by ACC

**FIGURE 3:**
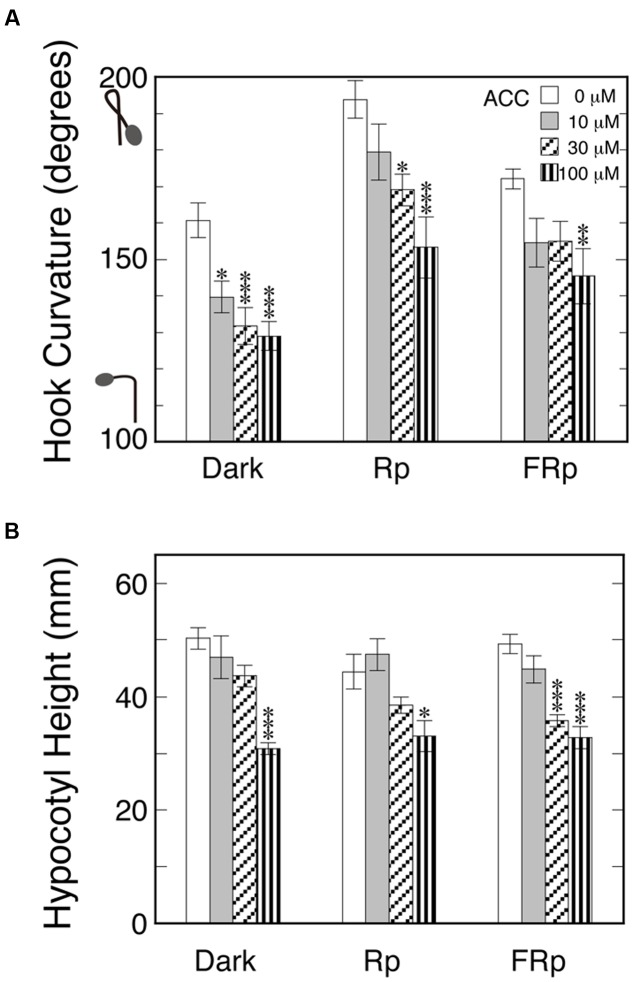
**Effects of ethylene precursor ACC on apical hooks formed in the dark and exaggerated by Rp or FRp (A)** and on hypocotyl height **(B)**. Five day-old dark grown seedlings were sprayed with 1-ml/bottle ACC solutions of the indicated concentrations or plane water, and 24 h later they were given 20-s Rp or 40-s FRp or no pulse, and then stood in the dark for further 48 h until the results were determined. The culture bottles were kept in the cap-loose mode throughout the experiment. Rp: R_LED_, 193 μmol m^-2^ s^-1^; FRp: FR_LED_, 465 μmol m^-2^ s^-1^. Histogram bars: mean ± SE (*n* = 10–29); statistically significant differences, respectively, at ^∗^*P* < 0.05, ^∗∗^*P* < 0.005, ^∗∗∗^*P* < 0.0005 as compared with the null ACC control within each light condition. cv. Sekaiichi.

Endogenous ethylene may give some different effect by evolving differently among the target tissues of seedlings; for example, between the concave and convex sides of the hook. To examine this possibility, seedlings were sprayed with various concentrations of ACC, the immediate biosynthetic precursor of ethylene, and kept in the dark for 72 h. To minimize the accumulation of emitted ethylene, the bottles were kept in the cap-loose mode. As the standard for comparison of the expected effects of ACC, Rp or FRp was given to induce hook exaggeration not only in the absence of ACC, but also in the presence (**Figure 3A**). The hook angles presented themselves comparatively small due to the pulse of light here compared with those of Rc and FRc in **Figure 2A**. All the three sorts of hook curvatures including the one kept in the dark throughout were similarly suppressed by ACC concentration-dependently, and excluded the above-stated assumption that endogenously evolving ethylene may cause hook exaggeration instead of light. The effects of the ACC on hypocotyl height (**Figure 3B**) did not differentiate the treatments Dark, Rp and FRp, supporting that ethylene evolved from ACC equally regardless of the light pulse given and operated normally in this test system.

### Effect of Continuous Red and Far-Red Light on Ethylene Evolution from ACC

Although it was suggested in **Figure 3B** that R and FR do not affect ethylene evolution from ACC, it was indirect proof with a single pulse of light. Accordingly, the same question was examined by direct quantification of ethylene evolved from ACC under Rc, FRc and in darkness. How high a concentration the evolved ethylene reached in the bottles was another important question to be answered.

**FIGURE 4:**
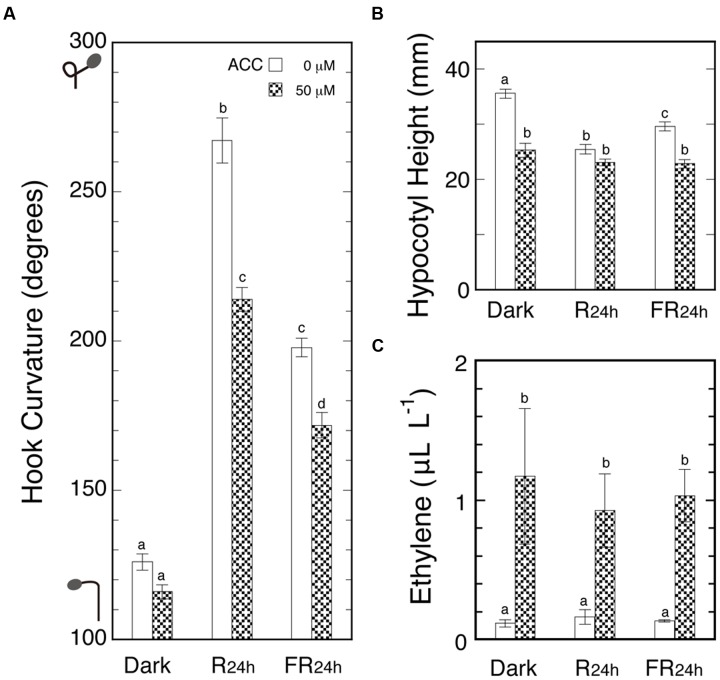
**Effects of ACC on apical hooks in the dark and under continuous 24-h R or FR (A)**, hypocotyl height **(B)** and ethylene concentrations in culture bottles **(C)** at the end of experiment. After grown in the dark in the cap-loose mode for 5 days, seedlings were sprayed with 1-ml/bottle of 50 μM ACC solution or plain water, and immediately shifted to the cap-tight mode and kept for 48 h until the results were determined. The 24-h R and FR started at the time of the shift to cap-tight mode. Rc: R_FL_, 50 μmol m^-2^ s^-1^; FRc: FR_FL_, 23.5 μmol m^-2^ s^-1^. Histogram bars: mean ± SE (*n* = 40–50) for **(A)** and **(B)**, and mean ± SD (*n* = 4–5 bottles) for **(C)**. Different letters of a, b, c, and d on the top of bars show pair-wise statistically significant differences at *P* < 0.05 within each Figure. cv. Ponte-Rosa.

Seedlings were sprayed with ACC solution or water and cultured in the cap-tight mode for 48 h with or without R_24 h_ or FR_24 h_. To magnify the effects of light, the irradiation continued for 24 h starting at the ACC spray, and as the result, marked light-induced hook exaggeration (LIHE) and hypocotyl growth inhibition were caused (**Figures 4A,B**). But the ethylene evolution did not differ among the above three conditions, giving the almost identical final concentrations in the neighborhood of 1.0 μL L^-1^ (**Figure 4C**). The concentration range corresponded with that which suppressed the hook curvatures most effectively not only in the dark, but also under Rc or FRc (**Figure 2**).

Thus, the possibility is excluded that endogenous ethylene evolving from its immediate precursor ACC by a possible light action might cause LIHE. It was also confirmed that ethylene suppresses hook curvature in the neighborhood of 1.0 μL L^-1^.

### Effect of Reduced Ethylene Emission on Hook Curvature

**FIGURE 5:**
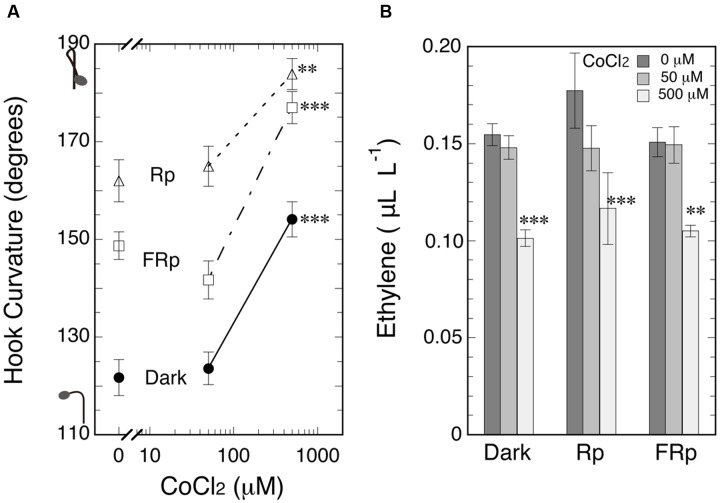
**Effects of ethylene biosynthesis inhibitor CoCl_2_ on hook curvatures exaggerated by Rp or FRp and on the dark control (A)**, and the ethylene concentrations in culture bottles **(B)**. After grown from seeds on CoCl_2_ solutions (0, 50, and 500 μM) in the dark, cap-tight mode for 5 days, seedlings were irradiated with an Rp or FRp and cultured for further 46 h in the dark, cap-tight mode. Rp: R_LED_, 193 μmol m^-2^ s^-1^ for 20 s; FRp: FR_LED_, 465 μmol m^-2^ s^-1^ for 40 s. Data points: mean ± SE (*n* = 20–30) for **(A)**, and mean ± SD (*n* = 3 bottles) for **(B)**. Statistically significant differences, respectively, at ^∗∗^*P* < 0.005, ^∗∗∗^*P* < 0.0005 as compared with the respective null CoCl_2_ control. cv. Seiko No.17.

What will happen to hook curvatures, when endogenous ethylene level is reduced below normal? To reduce endogenous ethylene level, the ethylene biosynthesis inhibitor CoCl_2_ was firstly tested. Seedlings were grown from seeds on CoCl_2_ solutions of various concentrations including null concentration in the cap-tight mode for 5 days, and an Rp or FRp was given to induce hook exaggeration (**Figure 5A**). As the result, hook curvatures of the irradiated and non-irradiated seedlings were equally enhanced by the inhibitor at 500 μM, and the ethylene levels were conversely suppressed at the same concentration. There was no significant difference in the reduced ethylene levels among the three different light treatments (**Figure 5B**).

**FIGURE 6:**
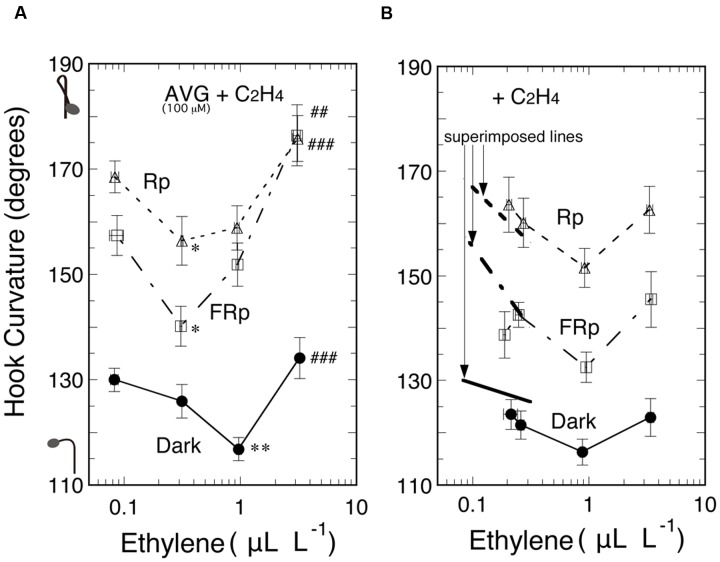
**Effects of supplemented ethylene on hook curvatures exaggerated by Rp or FRp and on the dark control in the presence (A)** or absence **(B)** of AVG. The thick lines superimposed for comparison in the low ethylene concentration area in **(B)** are excerpts from the corresponding three curves in **(A)**. After grown from seeds on 100 μM AVG solution in the dark, cap-tight mode for 5 days, seedlings were exposed to various concentrations of ethylene, irradiated with an Rp or FRp, and cultured for further 48 h in the dark until the resulting ethylene concentration in the bottles and hook angles were determined. The ethylene concentrations determined were used to plot the relevant hook angles. Supplemented ethylene: null, 0.3, 1.0, and 3.0 μL L^-1^ **(A)**; null, 0.3, 0.9, and 3.0 μL L^-1^ **(B)**; data points: mean ± SE (*n* = 20–30). Statistically significant differences, respectively, at ^∗^*P* < 0.05, ^∗∗^*P* < 0.01 as compared with the hook angle at the left end of each curve; ^##^*P* < 0.01, ^###^*P* < 0.0005, respectively, as compared with the valley of each curve in **(A)**. Rp: R_LED_, 193 μmol m^-2^ s^-1^ for 20 s in **(A)** and for 15 s in **(B)**; FRp: FR_LED_, 465 μmol m^-2^ s^-1^for 40 s in **(A)** and for 30 s in **(B)**. cv. Seiko No.17.

Secondly, another ethylene biosynthesis inhibitor AVG was sprayed to seedlings at null, 10, 100, and 500 μM. At 100 and 500 μM it enhanced the hook curvatures under the three light conditions to similar extents (data not shown). Next, seedlings were grown from seeds on 100 μM AVG for 5 days, and then supplemented with various concentrations of ethylene and irradiated with Rp or FRp, or not irradiated, followed by culture for 48 h (**Figure 6A**). Another similar experiment made without AVG gave the results as shown in **Figure 6B**. In both Figures the left ends of the curves indicate the concentration of only ethylene emitted by seedlings. Comparison of the left ends of corresponding curves between the two Figures indicates that the AVG treatment reduced endogenous ethylene from about 0.2 μL L^-1^ (also seen in **Figure 2A**) to about 0.08 μL L^-1^. For easy comparison of the effect of endogenous ethylene at reduced concentrations, excerpts from the three curves in **Figure 6A** are overlaid as the thick lines on the corresponding curves in **Figure 6B**. The excerpts fell, respectively, on the extensions of the corresponding curves obtained in the absence of AVG. Thus it is clear that AVG suppressed ethylene evolution, and, in turn, resulted in the enhancement of the hook curvatures, whether exaggerated or non-exaggerated, at the low concentration range below 1.0 μL L^-1^, and the enhancement was reversed by supplementing ethylene. These data confirm that ethylene suppresses all of the three kinds of hook curvature at the low concentration range.

At the high ethylene concentration range above about 1.0 μL L^-1^, on the other hand, where it tended to enhance the hook curvatures, the hook enhancement is obviously greater when fed with AVG (**Figure 6A**, compared with **Figure 6B**, also **Figure 2A**). This tendency seems more marked when irradiated with Rp or FRp than when kept in the dark, shifting the valleys of the curves for Rp and FRp to a lower ethylene concentration. Since the action of AVG as ethylene synthesis inhibitor is canceled by the supplemented ethylene, the hook enhancement found here is considered to be due to another unknown action of AVG.

### Effect of Auxin Polar Transport Inhibitor NPA on Hook Curvatures

Literature shows that an apical hook is formed by lateral localization of auxin at the apical part of the hypocotyl or epicotyl, and ethylene exerts its hook-exaggerating action through enhancing the localization of auxin (Vandenbussche et al., 2010; Žádníková et al., 2010, 2015; Abbas et al., 2013; Mazzella et al., 2014; Willige and Chory, 2015). It has also been known that auxin induces ethylene evolution in tissues (Kang et al., 1967; Kang and Ray, 1969; Yip et al., 1992; Schwark and Bopp, 1993; Abel et al., 1995; Peck and Kende, 1995; Coenen et al., 2003; Tsuchisaka and Theologis, 2004). Hence the present authors assumed that LIHE may also involve auxin localization intensified by light. To examine this possibility, effects of the auxin polar transport inhibitor, NPA, on hook curvature and ethylene emission rate were examined.

**FIGURE 7:**
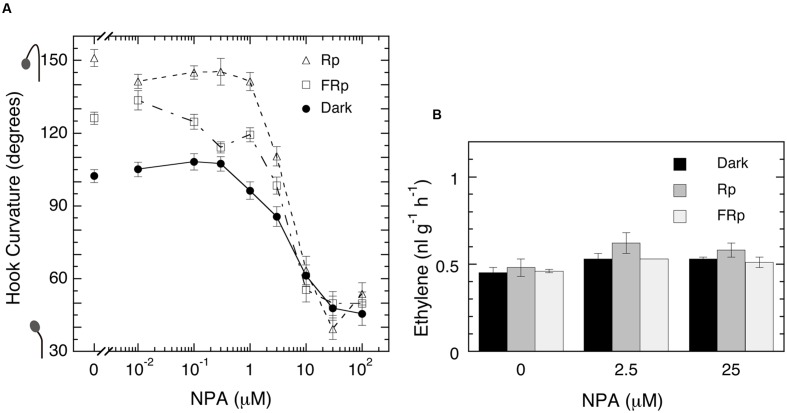
**Effects of auxin polar transport inhibitor NPA on hook curvatures exaggerated by Rp or FRp and the one formed in the dark (A)**, and ethylene emission rate of seedlings **(B)**. **(A)** After grown in the dark, cap-loose mode for 5 days, seedlings were sprayed with 1.3-ml/bottle NPA solutions of various concentrations, 12 h later irradiated with an Rp or FRp or non-irradiated, and stood in the dark, cap-loose mode for further 48 h. **(B)** After grown from seeds on NPA solutions in the dark, cap-tight mode for 5 days, seedlings were irradiated with an Rp or FRp and cultured for further 50 h in the dark until ethylene emission rates were determined. Non-irradiated controls were similarly cultured for the same periods in the dark throughout. Rp: R_LED_, 127 μmol m^-2^ s^-1^ for 20 s; FRp: FR_LED_, 552 μmol m^-2^ s^-1^ for 40 s. Data points: mean ± SE (*n* = 27–40) for hook angle **(A)**, and mean ± SD (*n* = 3) for ethylene determination **(B)**. No statistically significant difference at *P* < 0.05 in the effect of NPA on ethylene emission. cv. Ponte-Rosa.

As shown in **Figure 7**, the inhibitor suppressed markedly and uniformly the hook curvatures in all lots, irrespective of Rp, FRp or no pulse, at the concentrations over 1 μM, half inhibition being at 5 μM, and at 30 μM the hooks were almost straightened up (**Figure 7A**). The emission rate of ethylene, however, was not affected at all the concentrations tested (**Figure 7B**). These results exclude the possibility that ethylene evolution is controlled by auxin and regulates LIHE in tomato. At the same time this experiment with NPA suggests that LIHE is controlled by localization of auxin, as already shown with the apical hook curvature formed in the dark (Abbas et al., 2013; Mazzella et al., 2014; Willige and Chory, 2015; Žádníková et al., 2015).

### Effect of R and FR on Ethylene Emission by Seedlings

**FIGURE 8:**
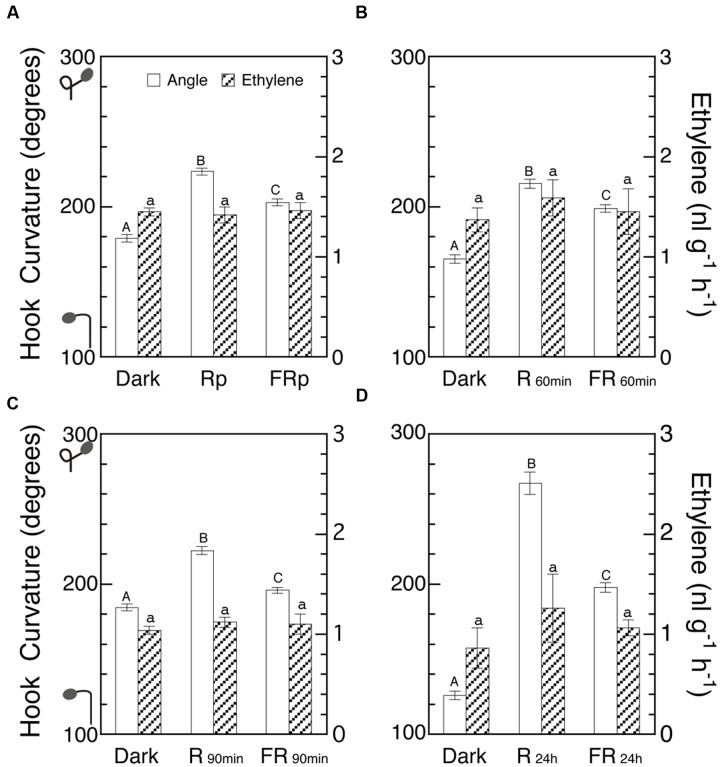
**Effects of various periods of R and FR on hook curvature and emission rate of endogenous ethylene.** After grown in the dark, cap-loose mode for 5 days, seedlings were shifted to the cap-tight mode, immediately irradiated with various periods of R or FR, and then grown in the dark for 48 h in total after the shift of mode until ethylene emission rate and hook angle were determined. Irradiation: **(A)** Rp, 10 s; FRp, 20 s; **(B)** 60 min each; **(C)** 90 min each; **(D)** 24 h each. Non-irradiated controls were similarly cultured for the same period in the dark throughout. **(A)**–**(D)** are of different runs of experiment. Rp: R_LED_, 193 μmol m^-2^ s^-1^; FRp: FR_LED_, 465 μmol m^-2^ s^-1^; R_60 min_ and R_90 min_: R_FL_, 33.8 μmol m^-2^ s^-1^; FR_60 min_ and FR_90 min_: FR_FL_, 21.9 μmol m^-2^ s^-1^; R_24 h_: R_FL_, 49.9 μmol m^-2^ s^-1^; FR_24 h_: FR_FL_, 23.5 μmol m^-2^ s^-1^. Histogram bars: mean ± SE (*n* = 40–77) for hook angle, and mean ± SD (*n* = 4–7) for ethylene emission rate. Different letters of A, B, and C on the top of bars for hook angles show statistically significant differences at *P* < 0.05 within each Figure. All bars for ethylene emission rate have only a, showing no significant difference. cv. Seiko No.17 for **(A)** and **(C)**; Sekaiichi, **(B)**; Ponte-Rosa, **(D)**.

If LIHE involves any variation of endogenous ethylene, the emission rate of ethylene by seedlings should be altered by R and FR. Some negative results to this assumption have already been described (**Figures 4** and **5**), but with more extensive coverage of irradiation periods it was examined whether ethylene emission rate is affected by R and FR according to the enhancement of hook curvatures (**Figures 8A–D**). Since the Figures represent the experiments on different dates, comparison of absolute values is possible only within each Figure, but for comparison among the Figures their ratios in each Figure should be used. Although the hook curvatures increased with the increasing duration of irradiation, the ethylene levels did not show any appreciable change, indicating that the emission rate of ethylene was not altered by the irradiations. This result is another piece of negative evidence against the possible involvement of ethylene as a limiting factor of LIHE.

## Discussion

The present study examined whether LIHE occurring in dark-grown tomato seedlings is mediated by ethylene, the evolution of which might be enhanced by light.

When applied exogenously to dark-grown 5-day old seedlings, ethylene suppressed (tend to open) hook curvature as its ambient concentration increased to about 1.0 μL L^-1^ and then turned to enhance it to some extent by its further increase up to 10 μL L^-1^, but at this concentration the toxicities of ethylene such as swelling at the subapical part of hypocotyl took place and did not allow the further growth of the hypocotyl and hence the additional enhancement of the hook curvature. The maximum hook curvature thus-caused by ethylene in the dark was far below the hook exaggerated by R or FR in the absence of supplemented ethylene (**Figure 2**).

Also, when supplied endogenously by application of the immediate precursor ACC, the ethylene failed to enhance hook curvature in the dark as well as under the action of Rp or FRp, but suppressed the hook curvature more markedly in proportion to the increasing concentration of ACC (**Figure 3**). This finding excluded the possibility that ethylene may evolve at different rates between the outer and inner sides of the hook, and causes differential growth of the hook part, resulting in the hook exaggeration.

The observed hook suppression by ACC is regarded as due to the suppressive action of low concentration ethylene (cf. **Figure 2A**), because the suppression of the hook was intensified with the increasing concentrations of ACC (**Figure 3**). In fact, the ambient concentration of the thylene evolved was only in the neighborhood of 1.0 μL L^-1^ even at as high a concentration of ACC as 50 μM (**Figure 4C**).

When the evolution of ethylene was suppressed by the biosynthesis inhibitor CoCl_2_ or AVG, LIHE caused by Rp or FRp was further enhanced and the enhancement was reversed by supplement of ethylene (**Figures 5** and **6**). Although, on the other hand, the auxin polar transport inhibitor NPA dramatically suppressed not only LIHE, but also the hook in the dark, it did not affect ethylene emission from the seedlings (**Figure 7**). Furthermore, no experiment showed that the emission of ethylene by seedlings was not altered by the irradiation of R or FR (**Figures 4C**, **5B**, **7B** and **8**), although if responsible for LIHE, the evolution of ethylene should be altered.

All of these results exclude the original assumption that endogenous ethylene may cause LIHE in 5-day-old dark-grown seedlings of tomato.

In contrast to the results described above, Chaabouni et al. (2009) and Gallie (2010) reported that the apical hook of dark-grown tomato seedlings was markedly enhanced by exposure to ethylene at the broad concentrations ranging from 0.1 through 10 μL L^-1^ without being suppressed anywhere in the concentration range. The photographs presented together in their papers show that the seedlings used there had already lost the seed coat and endosperm, and were at a more advanced stage than that of the seedlings used in our present study. At such an advanced stage we had previously noticed that no LIHE took place (Shichijo et al., 2010b; Shichijo and Hashimoto, 2013). Thus, the ethylene-induced hook exaggeration and LIHE are assumed to take place, respectively, at different developmental stages of dark-grown tomato seedlings. Besides, many other studies to report ethylene-induced hook exaggerations used as the materials *Arabidopsis* (Harpham et al., 1991; Lehman et al., 1996; Raz and Ecker, 1999; Knee et al., 2000; Raz and Koornneef, 2001; Li et al., 2004; Stepanova et al., 2008; Vandenbussche et al., 2010; Žádníková et al., 2010; Gallego-Bartolomé et al., 2011; Zhong et al., 2014), peas (Goeschl et al., 1967; Kang and Burg, 1972; Peck et al., 1998) or beans (Kang and Ray, 1969; Schierle and Schwark, 1988), and all of these plants belong to the group of species which exhibit no LIHE (Shichijo and Hashimoto, 2013). Hence, the present experimental results with dark-grown 5-day-old tomato seedlings do not contradict with the so far reported effects of ethylene on the apical hook.

What would then be possible as the mechanism of LIHE? Not only LIHEs induced by R or FR, but also non-exaggerated hook in the dark were strikingly suppressed by blocking the transport of auxin (**Figure 7**), indicating that the translocation of auxin plays a key role in the hook movements. It has been established that the apical hook in the dark is formed by differential distribution of auxin between the outer- and inner-sides of the hook part of the hypocotyl, mainly caused by PIN-FORMED (PIN), auxin-efflux carrier proteins, localized at the plasma membrane of cells there (Žádníková et al., 2010, 2015; Abbas et al., 2013; Mazzella et al., 2014; Willige and Chory, 2015). Phototropism, on the other hand, is also well known since Colodny and Went (Went, 1974) to be caused by lateral translocation of auxin toward the shaded side of the coleoptile or stem axis, and is enhanced by R-pre-irradiation through phytochrome action (Liscum et al., 2014). Haga and Sakai (2012) and Haga et al. (2014) demonstrated with *Arabidopsis* seedlings that R increases synthesis of PIN proteins, and reduces that of PINOID (PID) proteins, one of the subfamilies of AGCVIII kinases involved in PIN recycling (Willige and Chory, 2015) in the apical part of the hypocotyl, thus increasing the abundance of asymmetrically localized PIN proteins in the plasma membranes and, in turn, the asymmetrical distribution of auxin. This scheme for R amplification of phototropism is likely to be applicable to LIHE in tomato. Based on the findings with tomato seedlings that auxin transport plays the key role in the hook movement, it is speculated that R or FR regulates PIN and PID through phytochrome actions, resulting in an intensified accumulation of auxin at and growth inhibition of the inner side of the hook and, in turn, in exaggeration of the hook curvature.

The suppression of the apical hook curvature at the low concentration range (**Figure 2**) is an effect of ethylene discovered in the present study. It increases with increasing ambient concentration of ethylene up to about 1.0 μL L^-1^. It is noticed to occur in the dark as well as when the hook is exaggerated by R or FR (**Figures 2** and **6**). It was confirmed with endogenous ethylene supplied from its precursor ACC (**Figure 3**). Another proof is the effects of the ethylene biosynthesis inhibitors CoCl_2_ and AVG, which actually suppressed endogenous ethylene and enhanced (closed) the hook (**Figures 5** and **6**). The hook enhancement by lowering the concentration of or removing the endogenous ethylene is not great so as to explain LIHE, even if R or FR is assumed to reduce the level of endogenous ethylene. In any case it is interesting to note that the apical hook of young tomato seedlings as used in the present study is normally at the state somewhat suppressed (opened) by ethylene emitted by the seedlings themselves.

The suppression of the apical hook by low concentration ethylene was not so far found in *Arabidopsis* and other species, the apical hook of which was markedly enhanced by ethylene as referred to above, and is suspected to be related with the dull responsiveness of the hook in tomato to the hook-exaggerating action of ethylene known to occur extensively in other species. In any way the physiological significance of this newly found ethylene action is subject to future studies.

## Conclusion

In dark-grown 5-day old tomato seedlings, ethylene, whether endogenous or exogenous, does not enhance the apical hook enough to mimic LIHE. The ethylene emitted from seedlings is not altered by R or FR of fluences sufficient for LIHE. Thus, ethylene is not responsible. Instead, phytochrome-mediated promotion of laterally differential movement of auxin at the hook part is assumed to be most probable scheme for LIHE. At concentrations as low as about 1.0 μL L^-1^ or below ethylene suppresses (open) the apical hook in the dark as well as the ones exaggerated by R or FR. The apical hook curvature of dark-grown 5-day old tomato seedlings is normally at a state somewhat suppressed by endogenous ethylene.

## Author Contributions

MT-A performed experiments and data analysis. CS planned, guided and performed experiments, and wrote manuscript. ST provided and guided the use of gas-chromatograph. TH advised studies and wrote manuscript.

## Conflict of Interest Statement

The authors declare that the research was conducted in the absence of any commercial or financial relationships that could be construed as a potential conflict of interest. The reviewer C-KW and handling Editor declared their shared affiliation, and the handling Editor states that the process nevertheless met the standards of a fair and objective review.
